# Unraveling the Role of Flavor Structure and Physicochemical Properties in the Binding Phenomenon with Commercial Food Protein Isolates

**DOI:** 10.1021/acs.jafc.3c05991

**Published:** 2023-12-07

**Authors:** Cristina Barallat-Pérez, Hans-Gerd Janssen, Sara Martins, Vincenzo Fogliano, Teresa Oliviero

**Affiliations:** † Department of Agrotechnology and Food Science, Wageningen 6708 WE, The Netherlands; ‡ Unilever Foods Innovation Centre, Wageningen 6708 WH, The Netherlands; § AFB International EU, Oss 5342 LZ, The Netherlands

**Keywords:** flavor structure, protein-flavor binding, plant-based proteins, commercial food protein isolates, molecular interactions

## Abstract

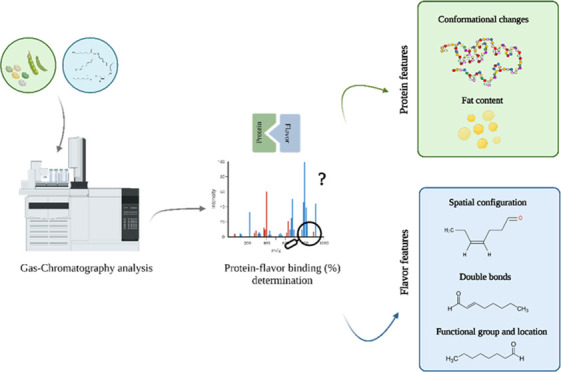

Food protein-flavor binding influences flavor release and perception. The complexity of the binding phenomenon lies in the flavor and protein properties. Thus, molecular interactions between commercial whey- or plant-based protein isolates (PI) such as pea, soy, and lupin, with carbonyl and alcohol flavor compounds were assessed by static headspace (HS) GC-MS. HS results showed that not only the displacement of the carbonyl group from the inner part of the flavor structure toward the edge promoted binding up to 52.76% ± 4.65 but also the flavor’s degree of unsaturation. Similarly, thermal treatment led to a slight increase in hexanal-protein binding because of possible protein conformational changes. Protein’s residual fat (<1%) seemed insufficient to promote significant flavor binding to PI. Despite the complexity of commercial food protein isolates, the results displayed that binding is predominantly influenced by the flavor structure and physicochemical properties, with the protein source and residual fat playing a secondary role.

## Introduction

Over the last decades, consumers’ food-related needs have gradually evolved to the search and consumption of more plant-based foods.^1^ Plant-based protein isolates (PI), such as proteins isolated from peas (*Pisum sativum* L.) (PPI) and soybeans (*Glycine max* L.) (SPI), emerged as substitutes to animal proteins in the development of novel plant-based protein foods (i.e., plant-based beverages) thanks to their high protein levels, low-fat content, and suitable techno-functional properties.^2^ Currently, lupin (*Lupinus angustifolius* L.) protein isolate (LPI) has gathered great interest to be used in high-protein-based beverage applications because of its low gelling and viscosity properties.^3^

From a molecular point of view, plant-based proteins structurally differ from animal-derived proteins. Plant-based proteins are usually seed proteins characterized by more complex tertiary and quaternary structures, higher hydrophobicities, and greater molecular weight. This is often accompanied by an enhanced abundance of nonpolar amino acids in the protein sequence.^4^ There exist significant structural disparities not only between animal- and plant-derived proteins but also among various plant-based protein sources. Although plant-derived proteins may seem structurally comparable to one another, the presence of intra- and interdisulfide bridges, α-helices, or β-sheets in their structure leads to a variability underpinning unique molecular interactions between the food elements (i.e., carbohydrates, fat, sugars, flavor compounds, etc.) present in the food system. In consumer studies, plant-based protein foods, including beverages, are often ranked as inferior in taste, texture, and appearance as compared to their animal counterparts.^1^ To tackle this issue and optimize the final food flavor profile, food developers often use flavor compounds as flavorings to improve the organoleptic characteristics of plant-based foods. Note that there are no homogeneous congruences easily found across the literature when referring to “flavor compounds”, “aroma compounds”, and “volatile organic compounds”. Therefore, to be consistent, this study will use the term flavor compounds.

The added flavor compounds are known to extensively interact with the plant-based proteins through physical or chemical molecular interactions.^5^ These interactions may result in reversible and weak bonds (i.e., hydrophobic interactions, hydrogen bonds, van der Waals forces, ionic/electrostatic forces) or nonreversible and stronger bonds (covalent linkages).^4,6^ Hydrophobic interactions typically entail the intricate interplay between the nonpolar hydrophobic interior of the protein and the nonpolar (aliphatic) segment within the flavor compounds (e.g., aldehydes and ketones). Likewise, aldehydes can be involved in chemical bonding via covalent linkages with proteins by reaction with the ε-amino group of lysine resulting in amide linkages.^4^ Conversely, hydrogen bonds tend to play a predominant role in the presence of aliphatic alcohols. It is then reasonable to assume that the complexity of protein-flavor binding strongly lies in the flavor’s molecular structure and physicochemical properties and closely impacts flavor perception. Nevertheless, when using commercial food protein isolates, the protein structure might not be overlooked.

Since the early 1950s, two-step extraction/isolation procedures have been used to industrially produce pea, soy, and lupin protein concentrates/isolates.^7^ Protein purity in isolates and concentrates depends on the separation method and starting substrate, resulting in different purity levels in terms of carbohydrates, fat, and sugars. However, most protein-flavor investigations use extracted, defatted, and highly purified proteins and/or protein fractions (i.e., β-conglycinin, glycinin, β-lactoglobulin, α-lactoglobulin, etc.),^8−10^ which are generally not used in food processing. Commercial and laboratory-purified proteins widely differ in structural, physicochemical, and techno-functional properties (i.e., rheological behavior, viscosity, gelling properties, water solubility, etc.).^11,12^ From a molecular perspective, an in-depth structural investigation of a single protein structure is necessary for a full understanding of the protein’s role in the flavor binding phenomena but may not have practical and realistic food applications. Currently, it seems unfeasible to achieve the requested requirements of food texture and appearance solely with isolated protein fractions. The meat and dairy analogues industry relies on the use of a mixture of nonrefined protein isolates to successfully meet the desired food standards. Despite their high protein content, fat residues may still be present, which is a factor of concern. Fat promotes the binding of flavor as these are mostly hydrophobic.^13^ From an industrial-applied perspective, the fat-flavor binding mechanism is generally considered to be a barrier during food formulation. The flavor profile of a food may be imbalanced, resulting in challenges in releasing and perceiving the flavor during consumption. Thus, the relative contribution of a protein’s residual fat on the protein-flavor binding mechanism should not be neglected.

Throughout food industrial processes, protein-based foods are held uninterruptedly for extended periods under different temperature conditions to prevent bacterial growth and ensure food product freshness and safety. The storage period between food manufacturing and food consumption may be lengthy, resulting in both food texture and food color changes, and in many cases, in deterioration of the food flavor and thereof, loss of food flavor quality.^14^ When thermal treatment is applied to further process the food product, protein structural modifications can occur^15^ as proteins may (partly) unfold and aggregate. Bread,^16^ coffee,^17^ and peanuts^18^ are some studied examples where time–temperature applied conditions were implemented to determine flavor binding. To the best of our knowledge, hardly any information is available concerning the impact of both time and processing temperatures on the flavor binding behavior of animal-based proteins, such as whey protein (WPI) and PI.

We hypothesize that the protein-flavor binding mechanism is mainly governed by the molecular structure and configuration. Thus, the present study aims to uncover the key role of flavor structure underlying the protein-flavor binding phenomenon with a special focus on the use of commercial food protein isolates (e.g., animal and plant-derived proteins). As flavor binding to proteins is a multifactorial rather than one-directional mechanism, the role of the flavor physicochemical properties may need to be considered as well. For this purpose, three PI (PPI, SPI, and LPI) and one animal-based protein (WPI), nonpurified and commercially available, were characterized utilizing spectrofluorimetric and NMR technology. Five aldehydes, four ketones, and one alcohol flavor compound were specifically selected to determine the influence of the unsaturation, spatial configuration, alkyl chain type, position of the carbonyl group, and chain length on the degree of binding and the binding mechanism. Flavor–matrix interactions were assessed by static headspace spectroscopy (HS) GC-MS.

The ultimate goal is to guide food manufacturers in food flavor creation and in efficiently designing novel plant-based food products based on consumer-desired flavor profiles while minimizing flavor dosing.

## Materials and Methods

### Materials

#### Flavor Compounds

The flavor compounds investigated were chosen based on their conformational and intrinsic physicochemical characteristics^19^ such as the unsaturation, spatial configuration, alkyl chain type, location of the carbonyl group, and chain length (Table S1). Hexanal, heptanal, *trans*-2-heptenal, *cis*-4-heptenal, octanal, 2-octanol, 2-heptanone, 2-octanone, 2-nonanone, and 2-decanone were purchased from Sigma-Aldrich (St. Louis, Missouri), and all had a purity of ≥95%.

#### Protein Sources

Plant-based protein isolates (PI) were acquired from different suppliers; soy protein isolate (SPI) Supro XT219D IP was kindly supplied by Solae (St. Louis, Missouri). Pea protein isolate (PPI) FYPP-85-C-EU was obtained from AGT (Waalwijk, The Netherlands), and lupin protein isolate (LPI) 10 600 was purchased from ProLupin (Grimmen, Germany). The animal-based protein used in this study, whey protein isolate (WPI) BiPro, was provided by Davisco International (Le Sueur, Minnesota). Manufacturer-specified specifications are shown in Tables S2 and S4. Nitrogen-to-protein conversion applied was *N* × 6.25. Proteins were selected based on their chemical structure, composition, and frequency of use in plant-based food alternatives (i.e., beverages). To decrease variability in the results, protein batches were kept away from light and oxygen and were adequately sealed and stored in a cool (10–15 °C) and dry place.

#### Other Chemicals or Materials

Na_2_HPO_4_·7H_2_O, NaH_2_PO_4_·2H_2_O, Na_2_HPO_4_, NaH_2_PO_4_·2H_2_, 8-anilino-1-naphthalenesulfonate, chloroform (99.8%), and methanol were of analytical grade and purchased from Sigma-Aldrich. Pierce BCA (HO_2_CC_9_H_5_N_2_) assay kits were acquired from Thermo Fisher Scientific, Inc. (Waltham, Massachusetts) and contained albumin standard ampules (2 mg/mL, 10 × 1 mL containing bovine serum albumin at a concentration of 2.0 mg/mL in 0.9% saline and 0.05% sodium azide), and two BCA (bicinchoninic acid) reagents: (A) Na_2_CO_3_, NaHCO_3_, (HO_2_CC_9_H_5_N)_2_ and C_4_H_4_Na_2_O_6_ in 0.1 M NaOH; (B) 4% CuSO_4_·5H_2_O (25 mL). Ellman’s reagent (5′,5′-dithio-bis (2-nitrobenzoic acid) (DTNB)) from Thermo Fisher Scientific, Inc. was used to estimate the protein sulfhydryl content (–SH). Tris-glycine buffer and ethylenediaminetetraacetic acid (EDTA) were obtained from Sigma-Aldrich.

### Methods

#### Preparation of Food Flavor Stock Solutions

Each of the selected flavors was separately prepared in a 100 mL amber bottle (Pyrex, Thermo Fisher Scientific, Inc.) and closed with a screw cap. Flavor stock solutions were made with sodium phosphate buffer (pH 7.0, 50 mM) at an initial concentration of 10 mg/L according to Wang and Arntfield.^15^ Flavor stock solutions were placed in an ultrasonic water bath (Elma Schmidbauer GmbH, Singen, Germany) for 1 h at 20 °C to ensure satisfactory dissolution of the flavor.

#### Preparation of Food Protein Solutions

Likewise, both animal and plant-derived protein solutions were created following an adapted version of the protocol by Wang and Arntfield^15^ using, respectively, each of the selected proteins (PPI, SPI, LPI, and WPI) at an initial concentration of 2 wv% in sodium phosphate buffer (pH 7.0, 50 mM). The protein content of 2 wv% was selected based on high-protein-based beverages available in the market. Subsequently, samples were vortexed for 10–20 s (3200 rpm, Genie II, Genie, Sigma-Aldrich) and placed into an ultrasonic water bath for 20 min at 20 °C to adequately mix the solutions. Next, protein solutions were repeatedly vortexed for an additional 10–20 s to guarantee a homogeneous dispersion of the mixture. To determine the effect of residual fat on the flavor binding to PI, PPI, SPI, and LPI were defatted (DPI, i.e., DPPI, DSPI, and DLPI) using an adapted version of Bligh and Dyer’s^20^ protocol. A solvent mixture of chloroform and methanol in a 1:2 v/v ratio was used. The product-to-solvent ratio was 1:9. Samples were vortexed for 5 min to allow for proper contact of the phases. Then, samples were centrifuged at 4700 g for 10 min (Multifuge X3R, Thermo Fisher Scientific, Inc.). Once all solvent was removed, samples were air-dried and stored overnight in a fume hood at room temperature (20–22 °C). The remaining fat was measured by using an NMR fat content analyzer (Oracle, CEM Corporation, Abcoude, The Netherlands).

#### Preparation of the Gas Chromatography–Mass Spectrometry Samples

From a 2 wv% protein solution, for each protein type, 1 mL was added into a 20 mL GC-MS vial followed by the addition of 1 mL of flavor stock solution. Thus, a final protein solution of 1 wv% and 5 mg/L flavor concentration was obtained. The reference sample was a buffered protein solution with no added flavors. The vials were then closed with a metallic screw cup and kept in a water bath (SW22, Julabo GmbH, Seelbach, Germany) at 30, 70, or 90 °C, respectively, shaking at 125 rpm for 3 h before headspace analysis. Samples were prepared in triplicate. After the preparation of these samples, they were stored at 5 °C and measured with a weekly frequency.

#### Binding Measurement and Calculation

Protein-flavor binding was assessed by HS through GC-MS (Agilent 7890A GC coupled to an Agilent 5975C with triple-axis detector MS, Agilent, Amstelveen, The Netherlands). The GC was operated in split mode 1:10 at 8 mL/min split flow. Samples were incubated and shaken for 14 min at 40 °C, following a modified version of the Wang and Arntfield^15^ protocol. Thereafter, 1 mL of sample headspace was injected. A DB-WAX 121–7023 column (20 m × 180 μm × 0.3 μm) run at 0.8 mL/min constant flow was used. The column temperature was programmed at a rate of 40 °C/min to 240 °C. Operating conditions for the mass spectrometer (MS) were 70 eV EI with a mass range between 35 and 200 Da. MassHunter Quantitative Analysis (MSD ChemStation F.01.03.2357) was used as the software for the quantitation of the flavor. Additionally, the NIST Mass Spectrometry Library (InChI Library v.105) was used to supply chemical and physical information about the selected flavor. Flavors were analyzed individually to avoid mutual competition for the protein binding sites. Flavor binding to proteins was calculated and expressed in %, in the absence and presence of protein, as depicted in eq 1(15)
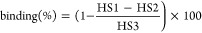
1where HS_1_ (protein solution + flavor) is the abundance in the headspace of the flavored-protein-based aqueous solution, and HS_2_ and HS_3_ are the abundances in the headspace in the absence of flavor (HS_2_) or protein (HS_3_).

#### Protein Surface Hydrophobicity

The surface hydrophobicity (*H*_0_) of PI and WPI was measured using an adapted version of the protocol described by Li-Chan, Nakai, and Wood.^21^ The *H*_0_ was determined using a spectrofluorometer (PerkinElmer LS50B, Thermo Fisher Scientific, Inc.). This measurement relies on the interaction between 8-anilino-1-naphthalenesulfonate (8-ANS) and the hydrophobic patches on the surface of the protein. Protein stock solutions were prepared in duplicate by mixing (Heidolph multi-Reax speed setting 9, Sigma-Aldrich, St. Louis, Missouri) for 4 h in 3.5 mg/mL sodium phosphate buffer (pH 7.0, 10 mM). After 4 h, the stock solution is centrifuged (Multifuge X3R, Thermo Fisher Scientific, Inc.) at 4700 rpm for 20 min. The protein concentration of the remaining solution was determined using a Pierce BCA protein assay kit according to the manufacturer’s instructions (Pierce, Thermo Fisher). After determination of the soluble protein concentration, a serial dilution was prepared in the range of 0.4–0.025 mg/mL protein. 25 μL of 8-ANS was subsequently added to 3 mL of each protein solution. The samples were left in the dark for 1 h to equilibrate. The fluorescence intensity (FI) of the samples was measured at an emission wavelength of 470 nm using an excitation wavelength of 390 nm.^22^ The *H*_0_ index was calculated as the slope of the plotted FI measurements vs concentration. The *H*_0_ was calculated from linear regression at a 95% confidence interval.

#### Protein Sulfhydryl Groups

Sulfhydryl groups (–SH) of PI and WPI were determined according to the adapted method of Ellman.^23^ Ellman’s reagent was prepared by dissolving 4 mg of DTNB reagent in 1 mL of tris-glycine buffer (0.086 M Tris, 0.09 M glycine, 4 mM EDTA, pH 8.0). Total and exposed –SH protein contents were obtained by suspending 3 mL of protein samples in 5 mL of reaction buffer and tris-glycine buffer with (total –SH) or without 8 M urea (exposed –SH), respectively. Then, 50 μL of Ellman’s reagent was added. The mixtures were incubated for 1 h at 95 °C in a water bath (SW22, Julabo GmbH, Seelbach, Germany) shaking at 125 rpm and then centrifuged (Multifuge X3R, Thermo Fisher Scientific, Inc.) at 12 000*g* for 10 min. The absorbance of the supernatant was determined at 412 nm with a reagent buffer as the blank. The exposed –SH contents (μmol –SH/g) were calculated by eq 2
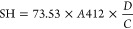
2where A412 is the absorbance at 412 nm, *C* is the protein concentration (mg/mL), and *D* is the dilution factor (considered 1 in the current study). The factor of 73.53 is derived from the molar extinction coefficient. Total –SH was calculated by adding the results obtained with and without urea.

#### Statistical Analysis

Data were computed and analyzed with Microsoft Excel and RStudio 4.2.1 (Boston, Massachusetts). Tukey’s test following the analysis of variance was implemented to determine significant differences with a level of *p* < 0.05. Letters in captions denote significant differences in protein-flavor binding. Treatments with the same letter are not significantly different.

## Results and Discussion

### Flavor-Related Factors Influencing the Binding Phenomenon with Commercial Food Protein Isolates

#### Influence of the Flavor’s Degree of Unsaturation and Spatial Configuration: The Case of 7-Carbon Chain Length Flavors

**Figure 1:**
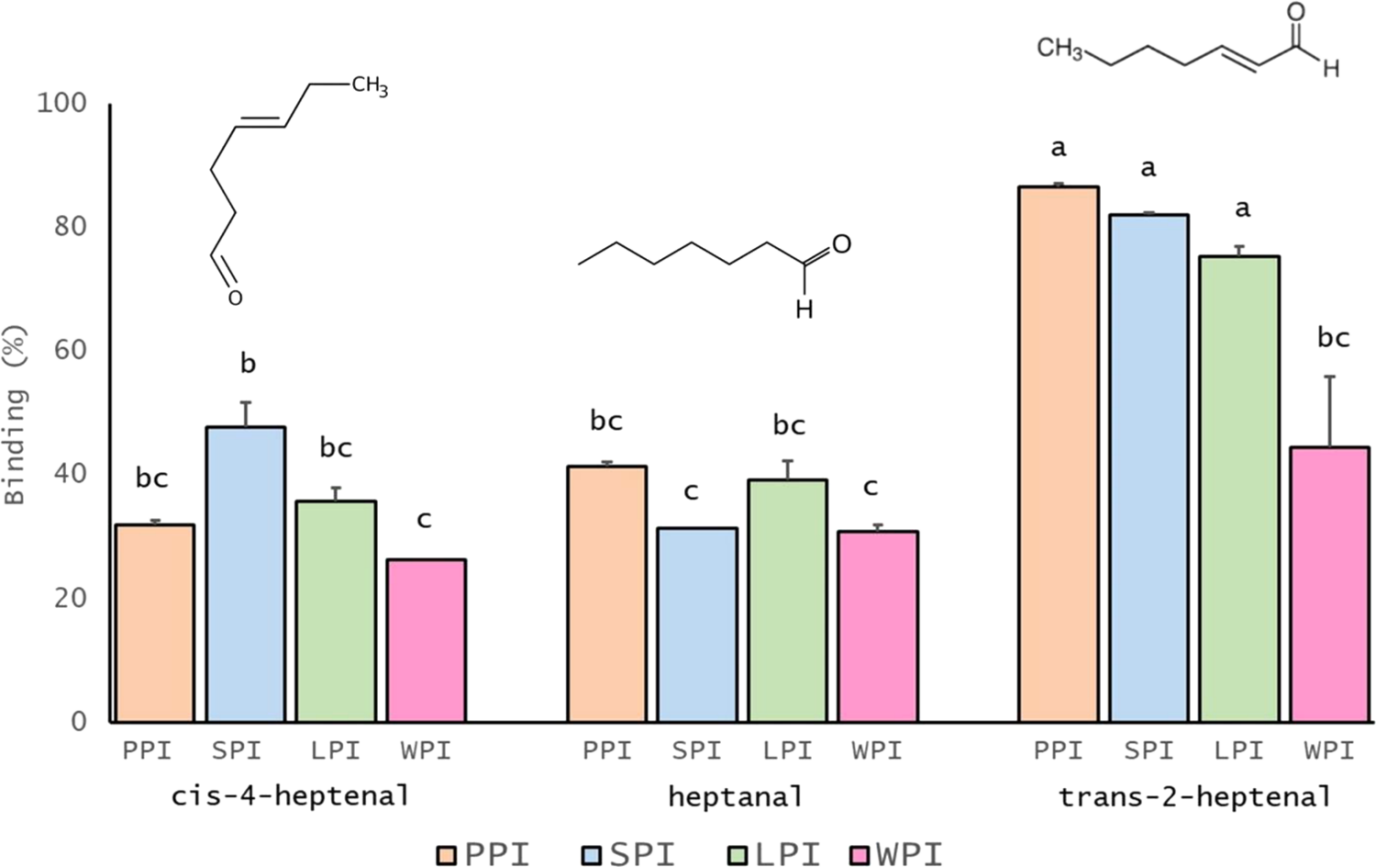
Influence of the flavor’s degree of unsaturation and spatial configuration on the binding phenomenon with commercial food protein isolates: *cis*-4-heptenal, heptanal, and *trans*-2-heptenal at 5 mg/L and protein isolates at 1%wv: pea (PPI), soy (SPI), lupin (LPI), and whey (WPI). Results are expressed as mean ± standard deviation. Letters denote significant differences (*p* < 0.05). Treatments with the same letter are not significantly different. Binding was calculated using eq 1.

To investigate the relationship between the flavor’s degree of unsaturation and the extent of the binding with PI and WPI, we compared *trans*-2-heptenal and heptanal (Figure 1). Figure 1 shows that the level of flavor binding to proteins increased with the unsaturation of the flavors. The addition of double bonds to the flavor chain, from heptanal to *trans*-2-heptenal, resulted in a significant binding increase from 13.73 to 54.60% ± 3.95 for PPI, SPI, and LPI. However, no significant differences were found for WPI. Note that occasionally a slight variability of data across repeated measurements of the same sample in independent measurements might be observed. As sample composition remains consistent across the replicates, the minor variation in the data might be because of small irregularities (e.g., sample carryover contamination) from the analytical instrumentation. However, possible inhomogeneity within the protein + flavor mixture should not be ignored.

The presence of a double bond in the flavor structure increases the molecular rigidity and electron density of the carbonyl group, thus enhancing protein binding.^24^ Unsaturation is majorly responsible for the compound’s structural stiffness^25^ and lack of flexibility to turn. Molecular rigidity promotes the exposure of the given functional group and, therefore, its propensity for interaction with the surrounding proteins.^24^ Additionally, *trans*-2-heptenal is 2 times less volatile than heptanal (Table S1) which may explain its stronger protein binding (Figure 1).

These findings are consistent with those of Zhou and Cadwallader^24^ and Kühn et al.,^26^ who evaluated the impact of the presence/absence of double bonds on the flavor structure and its binding effect on commercial food SPI and WPI, respectively. Similarly, the authors showed that *trans*-2-hexen-1-ol and *trans*-2-nonenal interacted more strongly with SPI and WPI than hexanal and nonanal. The closer proximity of the double bond and hydroxyl group (*trans*-2-hexen-1-ol) resulted in increased rigidity of the hydroxyl end of the molecule, facilitating the formation of hydrogen bonds with soy protein.^24^ Likewise, the occurring Michael addition or Schiff base reactions may imply strong covalent binding^4,26^ where the available double bonds react with lysine and histidine amino acids of the given protein.

The results obtained in the present section with commercial food protein isolates corroborated that the presence of double bonds plays a significant role in the binding phenomenon and seems to control the mechanism independently of the plant-based protein used. Interestingly, the comparison between plant- and animal-based proteins suggested that differences in protein architecture^3,27−34^ (Figure S1 and Table S3) may be a reason for different flavor binding affinity to *trans*-2-heptenal.

Based on the resulting binding similarity across PI, this information may help food developers to expand the use of alternative commercial food plant-based protein isolates in flavored-protein-based systems (e.g., meat and dairy alternatives) by tailoring flavor compositions based on the acquired knowledge regarding the importance of unsaturation on the flavor structure.

Nevertheless, the flavor spatial configuration and the resulting binding effect on PI and WPI are also considered and are shown in Figure 1. The change of flavor’s spatial configuration from spherical (*cis*-4-heptenal) to linear-shaped (*trans*-2-heptenal) significantly increased the protein binding from 18.19 to 54.60% ± 2.98 (Figure 1) despite the commercial food protein isolates used. Overall, across the studied proteins and under the specific experimental conditions applied, binding increased from *cis*-4-heptenal to *trans*-2-heptenal regardless of the proteins used.

Presumably, spherical-shaped flavors led to weaker binding to proteins compared to linear ones. The protein surface is characterized by “hydrophobic cavities” where small ligands can bind.^35^ Protein-flavor binding is partly governed by the specificity of the protein binding sites and the flavor stereostructure, where similar geometric shapes might fit precisely together.^36^ Therefore, as observed in Figure 1, the spherical shape of the flavor may potentially cause steric hindrance, blocking its access to the hydrophobic binding sites of the protein.

The relevance of the flavor spatial configuration on the protein binding phenomenon has been already pointed out and our results are in line with those of Zhou and Cadwallader,^24^ who noted that *cis*-3-hexen-1-ol (spherical-shaped) was retained to a smaller extent than 1-hexanol (linear-shaped) when studying commercial dehydrated SPI. The authors revealed that steric hindrance effects may lead to a decrease in accessibility to the hydrophobic binding sites on the protein, resulting in a reduction in the binding.

The binding of flavors to proteins is a multifactorial mechanism rather than a one-directional phenomenon, where the hydrophobicity and volatility of the flavor should not be overlooked. *Cis*-4-heptenal is considered more hydrophilic and volatile, and hence, a more polar compound than *trans*-2-heptenal, as log *P* < 2 (Table S1). The lower hydrophobicity and volatility of *cis*-4-heptenal explain its low binding ability as seen in Figure 1.

The obtained results with industrial protein isolates repeatedly confirm that flavor structure (i.e., unsaturation and spatial configuration) and physicochemical properties (i.e., hydrophobicity) appeared to influence binding more than did the source of protein. However, it is advisable to consider that the degree of flavor retention may be affected by the experimental conditions applied.^37^

#### Influence of Alkyl Chain Type and Location of the Carbonyl Group: The Case of 8-Carbon Chain Length Flavors

**Figure 2:**
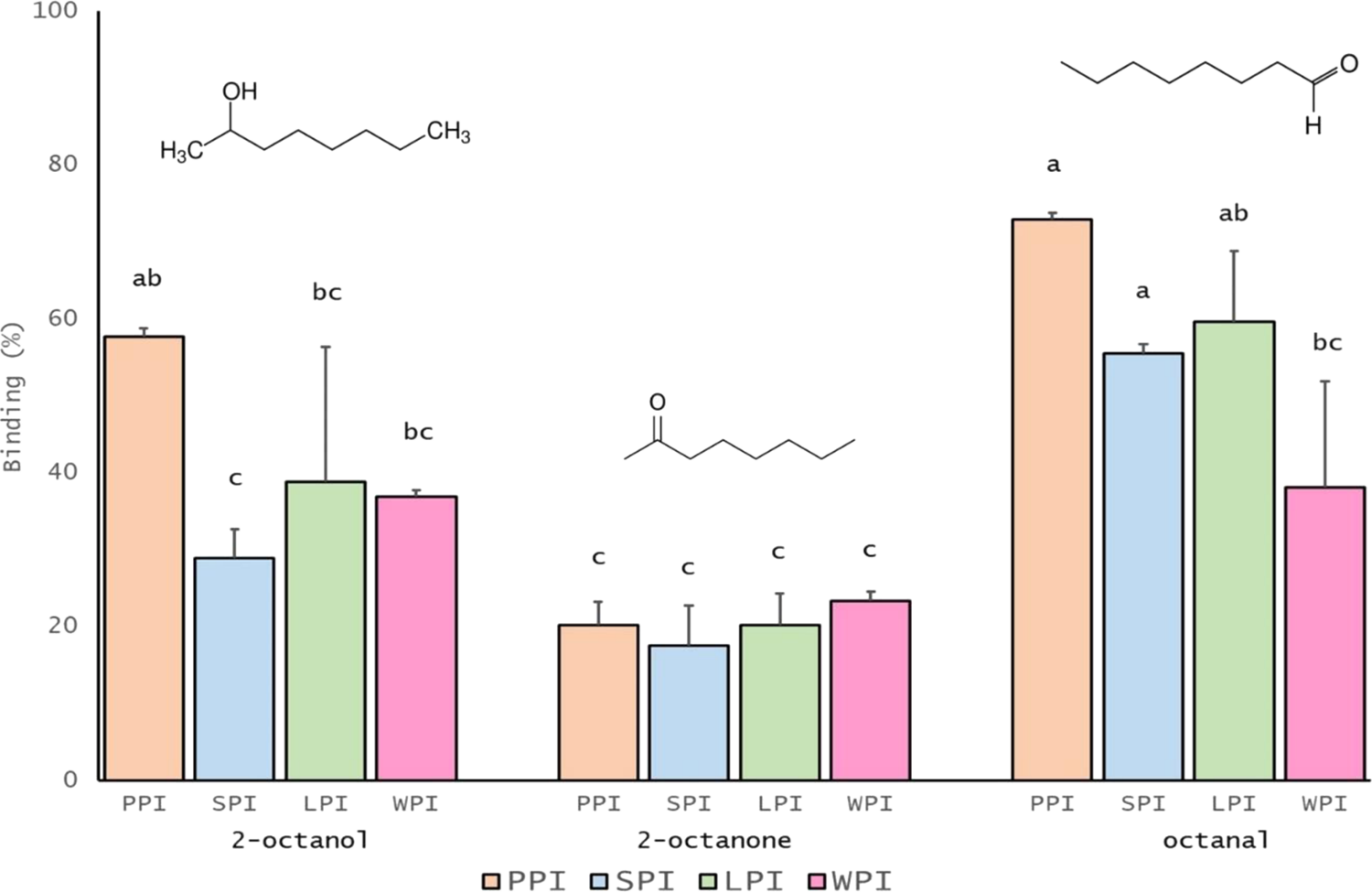
Influence of alkyl chain type and location of the carbonyl group on the binding phenomenon with commercial food protein isolates: 2-octanone, 2-octanol, and octanal at 5 mg/L and protein isolates at 1%wv: pea (PPI), soy (SPI), lupin (LPI), and whey (WPI). Results are expressed as mean ± standard deviation. Letters denote significant differences (*p* < 0.05). Treatments with the same letter are not significantly different. Binding was calculated using eq 1.

The alkyl chain type is hypothesized to have a substantial impact on the binding of the flavors to proteins. Therefore, to confirm this assumption, two flavors with the same carbon chain length (C8) and the same position of the radical group but different chemical functionalities and functional groups, such as 2-octanol (alcohol) and 2-octanone (ketone), were selected. Binding behavior across PI and WPI is summarized in Figure 2. As seen in Figure 2, flavor binding to commercial food protein isolates was found to be in the range of 28.85–57.7% ± 5.77 for 2-octanol and 17.53–23.30% ± 3.36 for 2-octanone, where significant differences were found for PPI.

Most of the alcohols are relatively hydrophilic; hence, they generally tend to show a weaker affinity to bind to the protein.^10,38^ However, the interaction between ketones and proteins is likely to be hydrophobic and thus show a stronger and higher binding affinity in an aqueous system.

Despite the existing role of flavor alkyl chain type in the retention with proteins, the hydrophobicity reflected by logP possibly suffices to explain the slight variations in binding affinity. A linear correlation is generally found between the hydrophobicity of the flavor and its binding affinity. Binding is increased when the flavor’s hydrophobicity increases.^39^ Presumably, 2-octanol is faintly more hydrophobic than 2-octanone (Table S1), which may explain the tendency for stronger retention across PI and WPI. Next to it, the higher boiling and melting point values of 2-octanol (Table S1) may provide comprehension of the resulting counterintuitive differences, as can be attributed to the strength of the hydrogen bonds. Hydrogen bonding facilitates intermolecular attraction, resulting in increased molecular adhesion. Consequently, a greater amount of thermal energy is needed to disengage these molecules, which in turn is reflected in high melting and boiling points.

Not only the alkyl chain type but also the location of the carbonyl group may play a role in the flavor binding mechanism. As observed in Figure 2, the studied ketone (2-octanone) is bound with a much lower affinity compared to that of the aldehyde (octanal). The presence of the carbonyl group located at the one end of the octanal molecule resulted in a larger binding as compared to the carbonyl group located in the middle of 2-octanone, regardless of the protein source. Generally, the displacement of the carbonyl group from the inner part of the molecule toward the edge leads to a significant binding increase from 14.73 to 52.76% ± 4.65 for the studies with plant protein isolates (PPI, SPI, and LPI). However, no significant differences were found for WPI. Overall, among the studied proteins and under the specific set of laboratory conditions applied in this investigation, binding increased from 2-octanone to octanal regardless of the protein used.

A polar keto group located at the end of the flavor structure is more easily accessible to establish interaction with the surroundings,^39^ and in this case with the protein hydrophobic pockets. If the keto group is found at the second position in the ketone structure, it may hinder hydrophobic flavor from binding to the proteins, lowering hydrophobic interactions and thus decreasing binding attraction. These results are aligned with the ones of Heng et al.,^9^ who studied the interactions of PPI fractions (legumin and vicilin) with aldehydes and ketones. Compared to the aldehydes, the ketones bind much less to vicilin, whereas no binding was observed between legumin and ketones. Damodaran and Kinsella^39^ reported that the free energy of association increases by 105 cal/mol for every move of the keto group from the terminal one position to the middle of the chain.

Protein-flavor interaction is primarily hydrophobic, but depending on the flavor chemical class chemical bonding via nonreversible covalent interactions may be present. Aldehydes, such as octanal, are known to react in Schiff base formation to establish covalent bonds with the ε-amino group of lysine residues^4^ resulting in amide linkages. Because of this interaction, leading to the observed lack of release, perception might be disrupted.

As seen before, the role of hydrophobicity seems crucial in unveiling the binding mechanism on protein matrices with flavors. Based on the hydrophobicity rule, octanal is slightly more hydrophobic than 2-octanone (Table S1), which may be causing stronger retention across the studied proteins.

It is worth noting that the examination of plant- and animal-based proteins has occasionally indicated that disparities in protein structure (i.e., differences in quaternary structure or sulfur-containing amino acids) (Figure S1, Tables S3 and S4) could potentially account for variations in their ability to bind with octanal.

### Commercial Food Protein Isolates: Factors Influencing the Binding Phenomenon with Food Flavors

#### Influence of Protein Residual Fat on the Protein-to-Flavors Binding Capacity: The Case of Ketones

The effect of protein residual fat content on the flavor binding was studied using a homologous series of ketones with increasing chain lengths. Ketones having 7 to 10 carbon atoms were selected based on their simple molecular structure which may also simplify the interpretation of the results. For this purpose, PI (PPI, SPI, and LPI) were defatted (DPI; DPPI, DSPI, and DLPI). WPI was not considered for the binding assessment as its non-defatted version already contained a negligible amount of fat (<0.05%). Manufacturer-specified and measured values of fat content before (non-defatted) and after (defatted) for PPI, SPI, and LPI are reported: For PPI: 8.3 wt % and DPPI: 2.46 wt %, for SPI: 3.1 wt % and DSPI: 1.10 wt %, for LPI: 3 wt % and DLPI: 0.11 wt %. Fat removal was conducted following the chloroform/methanol extraction protocol (see the Materials and Methods section).

**Figure 3:**
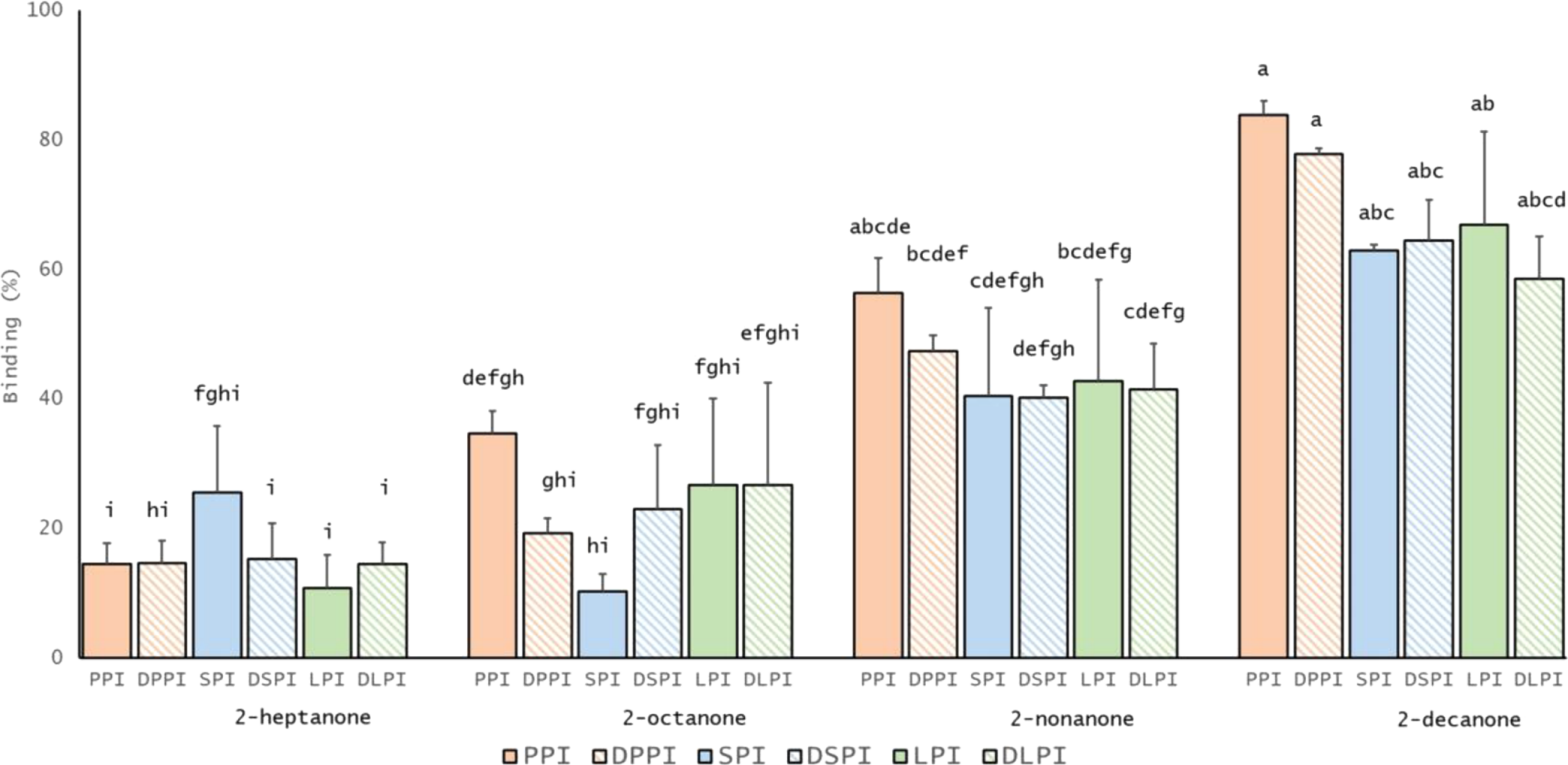
Influence of protein residual fat on the binding phenomenon with food flavors: 2-heptanone, 2-octanone 2-nonanone, and 2-decanone at 5 mg/L and protein isolates at 1%wv: pea (PPI), soy (SPI), and lupin (LPI). Non-defatted samples (PPI, SPI, and LPI) are the filled-colored columns, whereas the defatted samples (DPPI, DDSPI, and DLPI) are the stripped-colored columns. Results are expressed as mean ± standard deviation. Letters denote significant differences (*p* < 0.05). Treatments with the same letter are not significantly different. Binding was calculated using eq 1.

Figure 3 shows that all studied PI binds the selected volatile flavors. The extent of flavor binding increases with increasing flavor chain length and hydrophobicity. As seen in Figure 3, a slight increase in flavor binding affinity to PI can be noticed when increasing PI’s fat content. However, these differences were not statistically significant as treatments with the same letter are not significantly different. PPI had 15.38% ± 2.92, 8.9% ± 3.87, and 6.07% ± 1.53 greater binding to 2-octanone, 2-nonanone, and 2-decanone than DPPI, respectively. Similarly, the binding values for SPI to 2-heptanone and LPI to 2-decanone were 10.29% ± 7.95 and 8.34% ± 10.45 higher than compared to their defatted version, DSPI and DLPI (Figure 3). Unexpectedly, in DSPI and DLPI systems, 2-heptanone seemed to bind to a higher extent than when compared to non-defatted systems.

On the one hand, the increase in ketone-PI binding seen for the increased chain length (Figure 3) indicated hydrophobic interaction. This observation is in line with Damodaran and Kinsella^39^ who studied the interaction between SPI and ketones. These authors indicated that for each increment in the flavor chain length, the flavor binding increased accordingly, with a corresponding change in the free energy of about −600 cal/CH_2_ residue.^39^

On the other hand, even though not statistically significantly different, flavor binding to PI is slightly more visible in the non-defatted samples because of the lipophilic nature of the flavors (Figure 3). This effect becomes more noticeable when the lipophilicity of the flavors. These results are in agreement with those of Repoux et al.,^40^ who investigated the effect of fat content and flavor release on processed casein model cheeses. Comparably, higher binding for 2-nonanone was observed in high-fat cheeses (50%, fat content per dry matter) than in low-fat cheeses (25%) due to the strong hydrophobicity of the compound.^40^

PI are mainly composed of protein (>83%, Table S2). PPI contained the highest fat level (8.3 wt %) as compared to SPI and LPI (3.1 and 3 wt %, respectively) showed overall, to have the highest binding effect. This means that initially, 0.083 wt % fat was present in the solution of 1 wt % PPI. When defatting, the DPPI fat level dropped to 0.0246 wt %. Even though relatively higher binding was generally observed for PPI when compared to DPPI, the residual fat available (<1%) seemed insufficient to promote significant flavor binding to PPI (Figure 3). The direct effect of fat has already been evidenced between commercial WPI (0.017 wt %) and aldehydes.^41^ To verify the impact of residual fat available in the WPI, Weel^41^ added fat to obtain the same quantities as present in the WPI to the flavor solutions. According to Weel,^41^ the residual fat available in the WPI was considered to play only a secondary role in the aldehyde retention.

Flavor affinity for fat may seem to depend more on the lipophilicity of the flavor rather than on the protein’s residual fat content or protein source. Roberts, Pollien, and Watzke^13^ studied the effect of fat content on flavor release from milk-based emulsions. They observed that flavors widely differed in their affinity for the fat. A compound’s lipophilicity is inversely proportional to the required amount of fat to decrease its headspace concentration.^13^ For instance, polar compounds may need a greater quantity of fat to decrease their volatility than nonpolar compounds.

Notwithstanding the intricacy of commercial food PI, the obtained results suggested that protein source and residual fat (<1%) have little to no significant binding effect. Flavor-to-fat affinity seemed to be flavor hydrophobicity dependent rather than dependent on the protein’s residual fat level and/or PI source. These results raise awareness of the critical role of fat availability and content (when >1%) during food production of flavored plant-based food products and may not be neglected for a successful flavor release and perception of these food products.

As PPI, SPI, and WPI are widely used in food production as whole food ingredients, rather than as isolated highly purified protein fractions, the acquired knowledge and impact of the whole (nonpurified) protein isolate on the protein-flavor binding mechanism challenge is of practical significance in food formulations.

#### Influence of Processing Temperatures and Storage Time on the Protein-to-Flavor Binding Capacity: The Case of Hexanal

**Figure 4:**
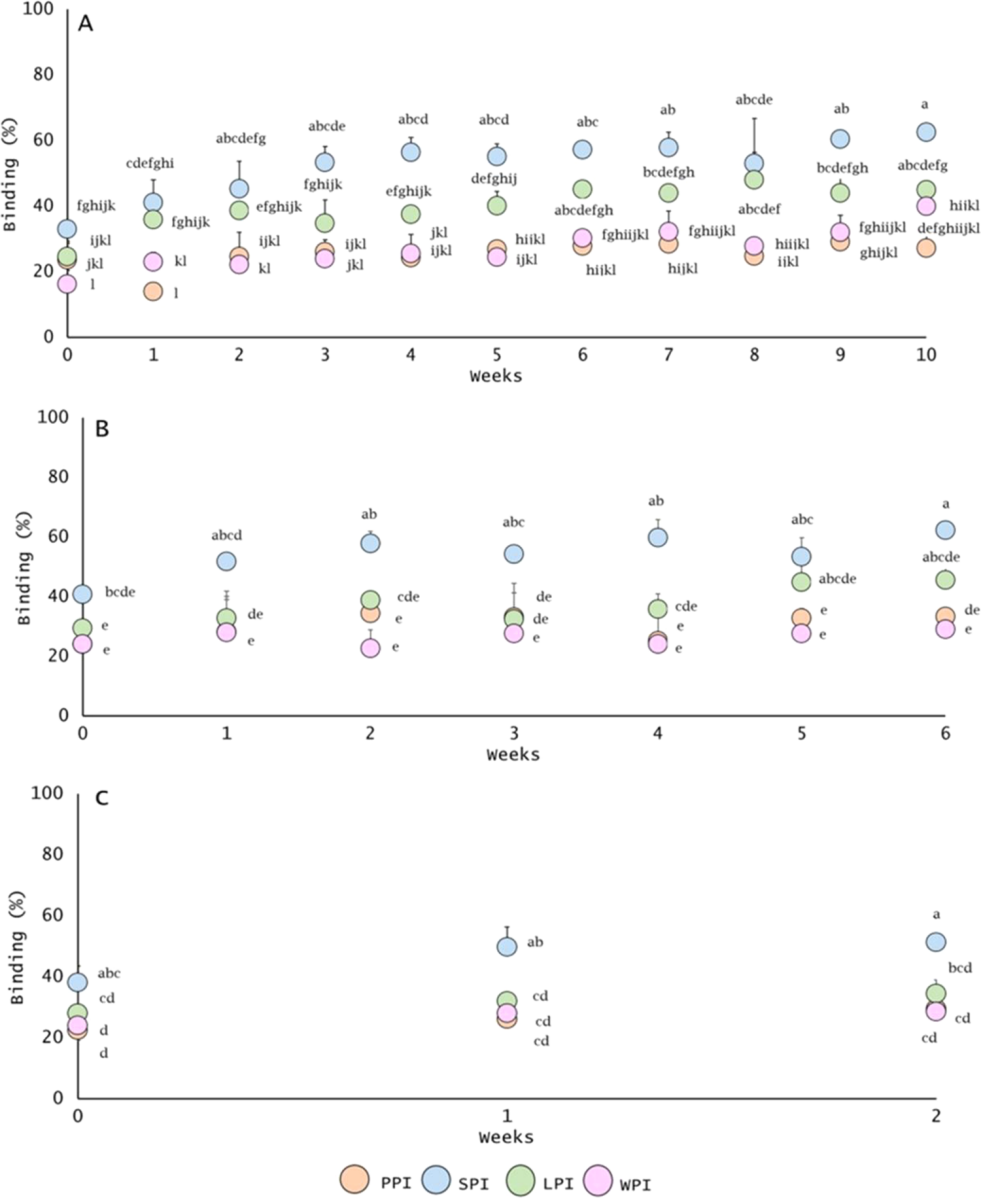
Influence of processing temperatures and storage time on the hexanal binding phenomenon with commercial food protein isolates: hexanal at 5 mg/L and protein isolates at 1%wv: pea (PPI), soy (SPI), lupin (LPI), and whey (WPI) during storage at 5 °C (A), 70 °C (B), and 90 °C (C). Results are expressed as mean ± standard deviation. Letters denote significant differences (*p* < 0.05). Treatments with the same letter are not significantly different. Binding was calculated by eq 1.

Headspace concentrations in different protein samples were examined to determine the effect of time and temperature conditions on the protein-flavor binding. For this objective, hexanal was selected because of its simple, straight-chain molecular structure. The flavored-protein-based aqueous solutions were stored at 5, 70, and 90 °C for several weeks to mimic refrigerated and elevated temperature storage conditions applied during industrial food processing. Results are shown in Figure 4. All measurements were performed in triplicate, with hexanal in the absence of a protein as a control.

During the first 3 weeks of storage, a slight but gradual increase in hexanal binding was found for SPI (Figure 4A–C), LPI (Figure 4B), and WPI (Figure 4A), regardless of the thermal treatment applied (5 and 70 °C). After the third week, hexanal binding reached a plateau (Figure 4A–C), where little or no further change in the hexanal binding behavior was observed. SPI exhibited a higher binding affinity for hexanal followed by LPI (Figure 4), independently of the temperature applied (5, 70, and 90 °C). In contrast, PPI and WPI showed the lowest binding affinity for hexanal. However, these differences were often not statistically significant. The variances in protein configuration (Figure S1 and Table S3) may account for minor differences in flavor binding.

Besides time, increasing the temperature also led to a slight increase in the level of hexanal binding (Figure 4). This observation possibly indicates that proteins might have undergone structural changes. Proteins are known to denature and (partly) unfold at temperatures above approximately 70 °C (72–84.5 °C).^42^ Structural changes in the protein can promote the exposure of the buried internal hydrophobic regions increasing their availability for flavor binding.^15^ These results concur with those of Hansen and Booker,^43^ who observed an increase in benzaldehyde binding to β-lactoglobulin when increasing temperature. Likewise, Wang and Arntfield,^15^ showed hexanal binding to canola proteins at 95 °C. Hexanal binding to canola proteins increased by ∼32% when applying heat. The authors suggested a strong affinity of hexanal for the disclosed binding sites exposed after canola protein unfolded during heat treatment. To corroborate these results and confirm the protein’s structural modifications because of temperature treatment, surface hydrophobicity (*H*_0_) was measured. Besides, the surface hydrophobicity probe assay, existing alternative methodologies to measure protein structural modifications were considered (e.g., circular dichroism spectroscopy, tryptophan fluorescence, etc.). However, for the current studied system containing commercial food protein isolates and no single protein fractions, surface hydrophobicity was proved to be particularly useful for monitoring structural changes from a mixture of proteins.^21^ Surface hydrophobicity reflects the number of hydrophobic groups on the surface of a protein molecule, and it is an excellent indicator of the protein properties and conformational changes.^21^ The *H*_0_ values of the studied protein are reported in Table 1. *H*_0_ was found to decrease by 21.82, 6.62, and 4.70% ± 9.38 for PPI, WPI, and SPI, respectively, after heat treatment (70 °C). When 90 °C was applied, the *H*_0_ decreased further up to 56, 27.67, and 24.26% ± 17.47 for LPI, SPI, and PPI, respectively.

**Table 1:** Experimental Values of the Surface Hydrophobicity (*H*_0_) of Pea (PPI), Soy (SPI), Lupin (LPI), and Whey (WPI) Protein Isolates at a 95% Confidence Interval

	*H*_0_ 5 °C	*H*_0_ 70 °C	*H*_0_ 90 °C
PPI	953 [649, 1257]	745 [505, 985]	564 [463, 666]
SPI	2010 [1605, 2415]	1916 [1541, 2291]	1385 [1188, 1582]
LPI	834 [752, 916]	1291 [1056, 1526]	566 [368, 765]
WPI	1692 [1585, 1797]	1579 [1245, 1914]	2374 [2303, 2446]

Protein oligomers can dissociate and reorganize themselves via hydrophobic forces to form high-molecular-weight aggregates when subjected to heat treatment.^42^ The slight decrease in H_0_ observed upon coagulation may suggest a partial implication of the hydrophobic residues in the aggregation process. Similarly, Li-Chan, Nakai, and Wood^21^ studied beef protein under thermal treatment. The authors reported that *H*_0_ dramatically decreased at 70 °C where coagulated meat particles were visible. The substantial reduction of *H*_0_ was attributed to the role of hydrophobic interactions in the aggregation and coagulation process, which facilitated protein structural changes. Upon heating at 90 °C, an increase in *H*_0_ for WPI indicated the exposure of hydrophobic regions incipiently concealed inside the protein core.^44^

Additionally, to further verify these conformational changes in proteins after heat treatment, changes in the exposed and total sulfhydryl content (–SH) of the proteins were measured (Table 2). The number of sulfhydryl groups in proteins is determined by the amount of sulfur-containing amino acids in the protein, i.e., methionine, and cysteine. In particular, nonheated and heated (>90 *°*C) conditions were compared. The data of exposed and total –SH content are shown in Table 2. As seen in Table 2, both exposed and total sulfhydryl content decreased after the protein had been heat treated. Among all of the studied proteins, SPI showed the highest –SH content.

**Table 2:** Experimental Values of Sulfhydryl Content (–SH (μmol/g)) of Pea (PPI), Soy (SPI), Lupin (LPI), and Whey (WPI) Protein Isolates Non-Heated (NH) and Heated (H)

	exposed –SH	total –SH
PPI_NH	3.98 ± 0.03	9.14 ± 0.24
PPI_H	2.88 ± 0.04	5.34 ± 0.086
SPI_NH	9.16 ± 0.19	15.62 ± 0.25
SPI _H	5.92 ± 0.15	11.54 ± 0.36
LPI_NH	7.13 ± 0.63	11.42 ± 0.64
LPI_H	4.19 ± 0.18	10.26 ± 0.24
WPI_NH	4.53 ± 0.02	8.78 ± 0.04
WPI_H	2.86 ± 0.08	6.39 ± 0.02

The decrease in the total and exposed –SH content is ascribed to alterations in the protein’s structure. Protein denaturation and unfolding lead to intra- or intermolecular thiol/disulfide (–SH/S–S) interchange or thiol/thiol (–SH/–SH) oxidation reactions.^45^ This occurring chemical reaction reduced the overall –SH content, which confirmed the proteins’ structural changes. This observation is in line with previous reports by Berghout^3^ and Jiang et al.^46^ The authors observed that heat treatment promoted a decrease in the amount of free sulfhydryl groups present in LPI and WPI, respectively, due to an oxidation and/or conversion of sulfhydryl groups into disulfide bonds. The obtained results proved that thermal treatment induced structural modifications of the protein (i.e., reduction of *H*_0_ and –SH contents), which consequently led to an increase in hexanal binding.

Exposed and total –SH quantified for SPI is higher than those for the other commercial protein isolates, as also expected from the relatively high amount of sulfur-containing amino acids in SPI (Table S4).

#### Influence of Commercial Food Protein Isolate Type on the Protein-to-Flavor Binding Capacity

Certainly, not all flavors are bound to the same degree to a given protein, and certain proteins may have greater binding capacity for some flavors than others. Among the PI, SPI generally showed the highest binding capacity to aldehydes (hexanal, heptanal, and *cis*-4-heptenal). Contrarily, WPI binds to aldehydes to a lesser degree (Figures 1 and 4). For ketones (2-heptanone to 2-decanone), PPI demonstrated the highest binding, followed by SPI and LPI (Figure 3). The differences in the quaternary structure between plant- and animal-based protein isolates may explain the occasional variable flavor binding patterns seen in this study. From a structural point of view, differences in a protein’s disulfide bond content might play a role in the binding mechanism (see Figure S1 and Table S3). WPI consists principally of globular proteins that have a tertiary structure stabilized by intramolecular disulfide bonds between cysteine residues (Figure S1 and Table S3).^32,33^ In contrast, PPI hardly contains disulfide bridges,^8^ thus allowing the flavor to readily interact with the hydrophobic sites of the protein, enhancing the binding. Additionally, there is a higher amount of disulfide bonds in WPI (Figure S1 and Table S3) may contribute to its lower flavor binding affinity (Figures 1, 2, and 4). Inter- and intradisulfide bridges bring stability to the protein structure. Increasing disulfide bonds on protein molecules may result in a more compact protein structure^47^ and accordingly, promote steric hindrance, blocking the access of small ligands such as flavors, and reducing flavor binding.

Based on the obtained results, flavor structure and intrinsic physicochemical properties principally contributed to protein binding. The presence of double bonds seemed to enhance flavor binding to PI and WPI more than in the absence of them. The degree of unsaturation of the flavor proved to govern flavor binding rather than the protein type. Likewise, the displacement of the carbonyl group from the inner part of the flavor structure toward the edge led to a significant binding increase. Contrarily, spherical-shaped flavors resulted in a lower binding degree compared to linear-shaped ones.

Flavor affinity to fat seemed to strongly depend mostly on the lipophilicity of the flavor rather than on the residual fat content present on the protein used. Therefore, when assessing the protein-flavor binding mechanism by using PI (>83% protein) defatting PI does not seem strictly required.

In the context of industrial food processing, it is imperative to consider the continuous exposure of protein-based food products to varying temperature conditions for prolonged durations. The obtained results showed that an increment in the temperature led to an overall slight increase in the level of binding of hexanal to the commercial food protein isolates. This observation possibly indicates that proteins might have undergone structural changes. Surface hydrophobicity and sulfhydryl content confirmed the idea of protein conformational changes, which caused stronger flavor binding. Despite the complexity of flavored-protein-based systems with commercial food protein isolates, the differences in flavor structure are achieved to explain the varied flavor binding patterns. The acquired outcome suggested that there is hardly any influence of the protein source and residual fat levels on the protein-flavor binding mechanism.

The above results may shed light on the fundamental mechanism of protein-flavor binding. Additional research may be necessary to explore a broader range of flavor chemical structures and intrinsic physicochemical properties, as the flavor structure has been found to have a significant impact on the binding phenomena. Most authors, when studying protein-flavor binding mechanisms, have investigated defatted, purified, and isolated protein fractions which are not realistic for use in food processing; instead, commercially available food protein isolates have more practical applicability. Accordingly, the rising awareness of the impact of industrial processing on protein structure by isolation techniques can provide valuable insights into the degree of denaturation of the starting protein isolate. This knowledge will assist food developers in enhancing the quality of flavored plant-based food products that incorporate industrially processed protein isolates.

## Supporting Information Available

Molecular structure of *Lupinus angustifolius* L, *Glycine max* L, *Pisum sativum* L, and *Lupinus angustifolius* L (Figure S1); physicochemical and structural features of the selected flavor compounds (Table S1); manufacturer-specified values of protein and fat content for pea, soy, lupin, and whey protein isolates (Table S2); molecular characterization of pea, soy, lupin, and whey protein isolates (Table S3); and manufacturer-specified values of amino acid content for pea, soy, lupin, and whey protein isolates (Table S4) (PDF)
