# Supplementary material for: Unraveling the Role of Flavor Structure and Physicochemical Properties in the Binding Phenomenon with Commercial Food Protein Isolates
Source: J Agric Food Chem. 2023 Dec 7;71(50):20274–84. doi: 10.1021/acs.jafc.3c05991 (PMC10739987; doi:10.1021/acs.jafc.3c05991)
Supplement: Supplementary file 1 — jf3c05991_si_001.pdf [file jf3c05991_si_001.pdf]

Unraveling the role of flavor structure and physicochemical properties in the binding phenomenon with commercial food protein isolates

Cristina Barallat-Pérez[a], Hans-Gerd Janssen[a,b], Sara Martins[a,c], Vincenzo Fogliano[a], Teresa Oliviero[a]

[a]Department of Agrotechnology and Food Science, Wageningen, The Netherlands; [b]Unilever Foods Innovation Centre, Wageningen, The Netherlands; [c]AFB International EU, Oss, The Netherlands.

Corresponding authors:

Cristina Barallat-Pérez (refereeing, publication, and post-publication)

e-mail address: cristina1.barallatperez@wur.nl

Tel: (+31) 317482520

Teresa Oliviero (post-publication)

e-mail address: teresa.oliviero@wur.nl

Tel: (+31) 624918793

SUPPORTING INFORMATION FOR PUBLICATION

Figure S1. Crystal structure of A) 7S conglutin δ (4PPH) from Lupinus angustifolius L., B) 7S Vicilin (7U1I) from Pisum sativum L., C) 7S β-conglycinin (1IPK) from Glycine Max L., D) bovine β-Lactoglobulin (5IO5) (RCSB)[27, 28, 29, 30, 31].

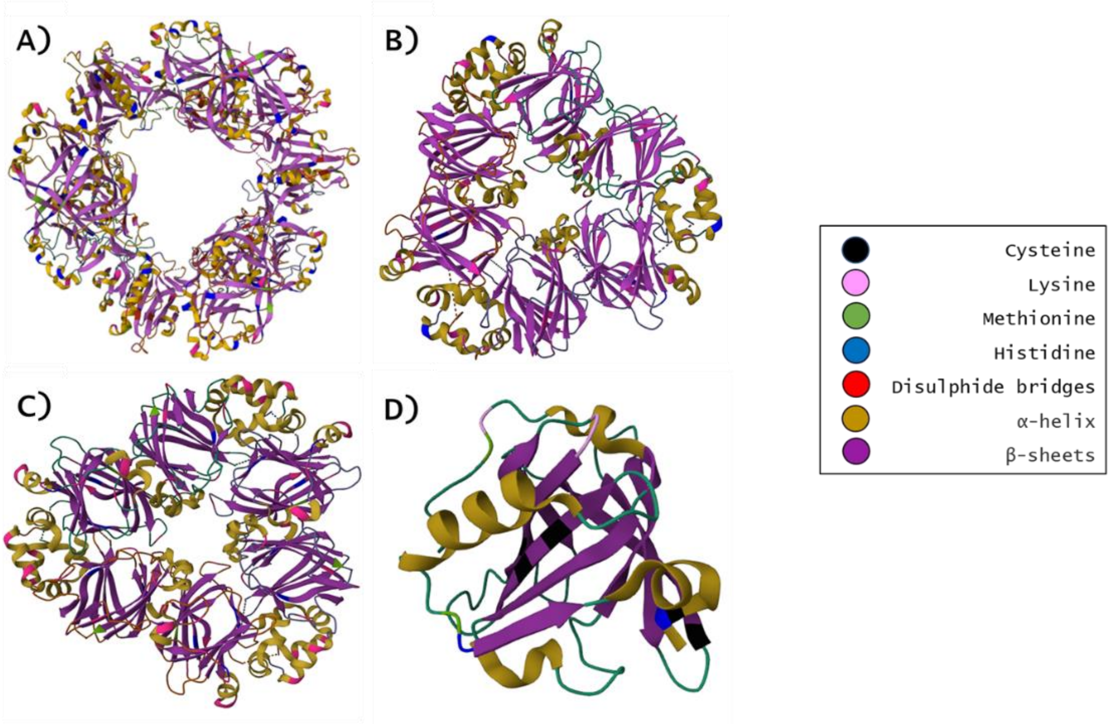

Table S1. Physicochemical and structural features of the selected flavor compounds.

Compounds	*CAS*	*Chemical structure*[1]	*LogP*[2]	*Molecular weight (g/mol)*[3]	*Vapor pressure (mmHg) at 25°C*[4]	*Melting point (°C)*[5]	*Boiling point (°C) at 760 mmHg*[6]
Hexanal ($C_6H_{12}O$)	66-25-1	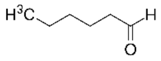	1.8	100.16	11.3	-58.2	130.0
Heptanal ($C_7H_{14}O$)	111-71-7	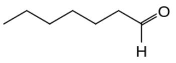	2.3	114.19	3.52	-43.3	152.0
Trans-2-heptenal ($C_7H_{12}O$)	18829-55-5	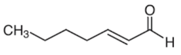	2.1	112.17	1.82	-53.35	166.6
Cis-4-heptenal ($C_7H_{12}O$)	6728-31-0	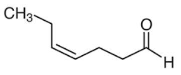	1.4	112.17	3.64	-53.35	151.6-160.0
2-heptanone ($C_7H_{14}O$)	110-43-0	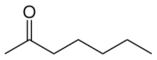	2	114.19	3.85	-35.5	151.5
Octanal ($C_8H_{16}O$)	124-13-0	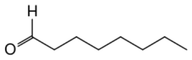	2.7	128.21	1.18	-23.0	171.0
2-octanol ($C_8H_{18}O$)	123-96-6	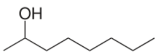	2.9	130.22	0.24	-31.3	179.0-181.0
2-octanone ($C_8H_{16}O$)	111-13-7	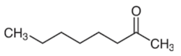	2.4	128.21	1.35	-16.0	173.0-175.0
2-nonanone ($C_9H_{18}O$)	821-55-6	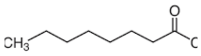	3.1	142.24	0.62	-150	193.5-198.0
2-decanone ($C_{10}H_{20}O$)	693-54-9	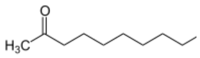	3.7	156.26	0.27	14.0	210.0

[1,2,3,4,5,6] *Computed properties obtained from PubChem (National Center for Biotechnology Information)*[19].

Table S2. Manufacturer-specified values of protein and fat content for pea (PPI), soy (SPI), lupin (LPI), and whey (WPI) protein isolates. Nutritional information per 100 g.

	Total fat (triglycerides) content	*Total protein content*
PPI	8.3%	79%
SPI	3.1%	91.2%
LPI	3%	91%
WPI	<0.05%	97.6%

**Values obtained from the supplier's raw material specification sheet.*

Table S3. Molecular characterization of pea (PPI), soy (SPI), lupin (LPI), and whey (WPI) protein isolates[3, 32, 33, 34].

	PPI			*SPI*			*LPI*				*WPI*	
Protein fraction	11S legumin	7S vicilin	2S convicilin	11S glycinin	7S β-conglycinin	2S	11S conglutin α	7S conglutin β	7S conglutin γ	7S conglutin δ	α-lactoglobulin	β-lactoglobulin
Molecular weight (kDa)	300-400	150-170	290	300-360	150-200	8-22	330-430	143-260	200	13	14	18.4
Disulfide bridges	6	0	N/D[1]	2	0	0	6	0	2	4	4	2

[1]*N/D= no available data*

Table S4. Manufacturer-specified values of amino acid content for pea (PPI), soy (SPI), lupin (LPI), and whey (WPI) protein isolates. Sulfur-containing amino acids per 100 g.

	Methionine	*Cysteine*	*Lysine*	*Histidine*
PPI	1.16%	1.23%	6.34%	2.04%
SPI	1.3%	1.3%	6.3%	2.6%
LPI	0.5%	1.2%	3.9%	2.3%
WPI	2.3%	2.8%	10.2%	2.0%

**Values obtained from the supplier's raw material specification sheet.*
